# Hemoglobin and human longevity: integrating oxygen transport, redox biology, and aging pathways – a narrative review

**DOI:** 10.1097/MS9.0000000000004508

**Published:** 2025-12-18

**Authors:** Emmanuel Ifeanyi Obeagu

**Affiliations:** a Department of Biomedical and Laboratory Science, Africa University, Zimbabwe; b The Department of Molecular Medicine and Haematology, School of Pathology, Faculty of Health Sciences, University of the Witwatersrand, Johannesburg, South Africa

**Keywords:** cellular senescence, erythropoiesis, hemoglobin, longevity, oxygen transport

## Abstract

Hemoglobin, the vital protein responsible for oxygen transport in the bloodstream, plays an indispensable role in sustaining life and cellular function. Traditionally viewed through the lens of hematology and respiratory physiology, hemoglobin is now being explored as a key determinant of healthy aging and human longevity. As the primary regulator of tissue oxygenation, its efficiency influences mitochondrial activity, energy metabolism, and organ vitality – all of which are central to lifespan regulation. Emerging evidence suggests that both low and excessively high hemoglobin levels are linked to increased morbidity and mortality in aging populations, underscoring the need for homeostatic balance. The dynamic interplay between hemoglobin concentration, erythropoiesis, hypoxia-inducible pathways, and oxidative stress reveals a complex biochemical network influencing aging at the molecular level. Hemoglobin’s oxidative byproducts, if unchecked, can induce cellular senescence, compromise immune responses, and contribute to degenerative conditions commonly associated with advanced age.

## Introduction

The pursuit of longevity has captivated scientists and clinicians for centuries, prompting the exploration of biological systems that regulate aging and age-associated diseases. Central to this exploration is the understanding of how oxygen, a molecule essential for life, is transported and utilized by the body. Hemoglobin, the principal oxygen-carrying protein in red blood cells, plays a critical role in sustaining aerobic metabolism by delivering oxygen from the lungs to peripheral tissues. Its function underpins cellular energy production, organ vitality, and systemic homeostasis – all factors intimately connected to human lifespan^[1–3]^. Hemoglobin’s significance extends beyond basic physiology; its levels and functional integrity are closely linked to health outcomes across the human lifespan. In elderly populations, deviations in hemoglobin levels – particularly anemia – are strongly associated with frailty, cognitive impairment, increased hospitalization, and mortality. On the other hand, abnormally high levels may predispose individuals to thrombosis and vascular complications. These observations suggest that hemoglobin serves as more than just a biomarker of oxygenation; it may be a critical regulator of longevity itself^[4–6]^. Aging is accompanied by a progressive decline in hematopoietic stem cell function, resulting in impaired erythropoiesis and altered hemoglobin production. These changes, often influenced by chronic inflammation, nutritional deficiencies, and hormonal imbalances, contribute to the pathogenesis of anemia in the elderly. The resulting tissue hypoxia can compromise mitochondrial efficiency and accelerate cellular senescence. Thus, preserving optimal hemoglobin function becomes essential in mitigating age-related decline and maintaining physiological resilience (Table 1)^[7–9]^.

**Table 1:** hemoglobin thresholds and their associated aging-related outcomes

Hemoglobin Level (g/dL)	Population	Associated Aging-Related Outcomes	Clinical Implications
< 12 (women), < 13 (men)	Older adults	Anemia; fatigue; cognitive decline; reduced exercise tolerance; increased risk of cardiovascular events	Nutritional assessment; iron, vitamin B12, folate supplementation; evaluate for chronic disease or renal impairment
12–14 (women), 13–15 (men)	Older adults	Optimal hemoglobin range; adequate oxygen delivery; preserved cognitive and physical function	Maintenance through balanced diet, regular monitoring, management of comorbidities
14–16 (women), 15–17 (men)	Older adults	High-normal range; generally favorable oxygen transport; potential increased blood viscosity in some cases	Monitor for cardiovascular risk, particularly in patients with hypertension or vascular disease
>16 (women), > 17 (men)	Older adults	Polycythemia; increased blood viscosity; elevated risk of thrombosis, stroke, and vascular complications	Assess for primary polycythemia, hypoxia, chronic lung disease; consider phlebotomy or cytoreductive therapy if indicated
HbA1c-associated glycated Hb > normal	Diabetic older adults	Impaired oxygen delivery due to glycation; enhanced oxidative stress; accelerated vascular and cellular aging	Glycemic control, antioxidant support, monitoring for microvascular complications

**HIGHLIGHTS**

- Hemoglobin levels critically influence tissue oxygenation, impacting aging and lifespan.
- Optimal hemoglobin balances oxygen delivery and oxidative stress.
- Low or high levels correlate with increased mortality.
- Genetic variants in hemoglobin affect longevity across populations.
- Targeting hemoglobin modulation offers therapeutic potential for healthy aging.

Moreover, the regulatory networks that govern hemoglobin synthesis are closely tied to adaptive mechanisms implicated in longevity. Hypoxia-inducible factors (HIFs), which regulate erythropoietin expression and hemoglobin production under low-oxygen conditions, are also known to modulate genes involved in angiogenesis, glucose metabolism, and cellular survival. Interventions that mildly activate HIF signaling – such as intermittent hypoxia, exercise, and pharmacological stabilizers – have demonstrated protective effects against aging-related degeneration, positioning HIF-hemoglobin pathways as promising targets in longevity research^[10]^. Oxidative stress presents another dimension through which hemoglobin may influence lifespan. As hemoglobin undergoes auto-oxidation, it produces reactive oxygen species (ROS), which, in excess, can damage DNA, proteins, and lipids, triggering pro-aging processes. Aging tissues typically show reduced antioxidant capacity, making them more vulnerable to ROS-mediated injury. Maintaining redox balance through antioxidant defense systems and preserving the functional integrity of hemoglobin is therefore crucial to cellular longevity^[11,12]^.

In addition to its role in oxygen transport, hemoglobin may also interact with various signaling pathways that influence inflammation, immune function, and vascular health. Chronic inflammation and immunosenescence are hallmarks of aging, and studies have shown that dysfunctional hemoglobin and heme overload can trigger pro-inflammatory cascades. Conversely, stabilizing hemoglobin structure and minimizing heme release may help modulate these pathways and contribute to healthier aging^[13,14]^. Lifestyle factors known to enhance longevity – such as regular physical activity, proper nutrition, and avoidance of environmental toxins – have been shown to positively influence hemoglobin levels and erythropoiesis. Exercise, for instance, stimulates erythropoietin production and promotes better oxygen utilization, while diets rich in iron, folate, and vitamin B12 support red cell formation. These observations reinforce the idea that hemoglobin status is not only a reflection of internal physiology but also a modifiable determinant of aging^[15]^.

## Aim

The aim of this review is to explore the multifaceted role of hemoglobin in the aging process, emphasizing its impact on oxygen transport, erythropoiesis, oxidative stress regulation, and tissue oxygenation.

## Methods

This narrative review was conducted to synthesize current evidence linking hemoglobin physiology, oxygen transport, and systemic aging processes with human longevity. A structured literature search was performed to ensure comprehensive coverage of mechanistic, clinical, and demographic perspectives relevant to the topic.

### Search strategy

Databases including PubMed, Scopus, Web of Science, and Google Scholar were systematically searched for peer-reviewed articles published up to September 2025. The search combined keywords and Medical Subject Headings (MeSH) terms related to hemoglobin, erythropoiesis, oxidative stress, tissue oxygenation, aging, longevity, and lifespan. Boolean operators (“AND,” “OR”) were used to refine the search. For example:

*“hemoglobin AND longevity”*

*“erythropoiesis AND aging AND oxidative stress”*

*“tissue oxygenation AND hemoglobin AND lifespan”*

### Inclusion criteria

- Original research, review articles, and meta-analyses addressing hemoglobin function in the context of aging and longevity.
- Studies reporting mechanistic insights into erythropoiesis, oxidative stress, or tissue oxygenation.
- Publications in English, focusing on human subjects or translational models relevant to human physiology.

### Exclusion criteria

- Studies with insufficient mechanistic detail or unrelated to hemoglobin physiology.
- Case reports or non–peer-reviewed articles lacking robust scientific evidence.

### Data extraction and synthesis

Relevant articles were screened for title and abstract, followed by full-text review. Key data extracted included study design, population characteristics, hemoglobin-related findings, aging outcomes, and mechanistic insights. Emphasis was placed on integrating molecular, physiological, and clinical perspectives to provide a cohesive narrative.

### Critical appraisal

While this review is narrative rather than systematic, methodological rigor was ensured by critically evaluating the quality, consistency, and relevance of included studies. Conflicting findings were discussed, and mechanistic pathways were synthesized to provide a comprehensive framework linking hemoglobin dynamics to human longevity.

### Ethical compliance

All sources were properly cited, and artificial intelligence-assisted drafting of this manuscript was conducted in accordance with the TITAN 2025 guidelines [Agha *et al* (2025)], with full transparency declared in the text.

### Hemoglobin: the oxygen lifeline

Hemoglobin is the quintessential oxygen-carrying protein in humans, functioning as the primary mediator of tissue oxygenation and cellular metabolism. Structurally, it is a tetramer composed of two alpha and two beta globin chains, each containing a heme group capable of binding one molecule of oxygen. This configuration allows hemoglobin to efficiently capture oxygen in the lungs and release it in peripheral tissues in response to local metabolic demand. The oxygen-carrying capacity of hemoglobin is finely regulated by allosteric modulators, including 2,3-bisphosphoglycerate (2,3-BPG), pH, and carbon dioxide concentration (Bohr effect), ensuring precise tissue oxygenation across diverse physiological states^[16,17]^. Beyond its classical role in oxygen transport, hemoglobin acts as a critical regulator of systemic homeostasis. Adequate hemoglobin levels support mitochondrial function and ATP generation, preserving cellular energy balance. Conversely, deviations from the optimal range – such as anemia or polycythemia – can disrupt oxygen delivery, precipitate oxidative stress, and accelerate cellular aging. Hypoxia induced by low hemoglobin activates HIFs, which trigger compensatory erythropoiesis but also promote inflammatory signaling and tissue remodeling, highlighting the delicate balance between oxygen supply and cellular resilience^[18,19]^.

**Figure 1.**
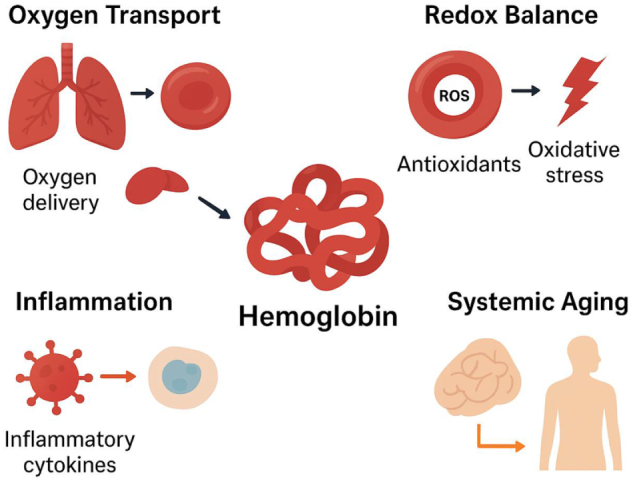
Hemoglobin’s Role in Oxygen Transport, Redox Balance, Inflammation and Systemic Aging.

Hemoglobin’s role extends into the maintenance of redox equilibrium. Heme groups can participate in reversible redox reactions, and the iron moiety within hemoglobin serves both as a cofactor for essential metabolic processes and as a potential source of ROS if unregulated. This duality underscores hemoglobin’s central position at the intersection of oxygen transport, oxidative stress, and longevity pathways^[20]^. Clinically, hemoglobin is a key biomarker of physiological aging and systemic health. Optimal hemoglobin concentrations are associated with enhanced cognitive function, cardiovascular resilience, and physical performance in older adults, whereas chronic anemia or elevated hemoglobin levels confer increased risk for morbidity and mortality. Furthermore, demographic factors such as sex, race, altitude exposure, and nutritional status modulate hemoglobin levels, emphasizing the importance of individualized assessment for predicting longevity outcomes (Fig. 1)^[21,22]^.

### Erythropoiesis and aging

Erythropoiesis, the process by which RBCs are produced, is central to maintaining optimal hemoglobin levels and ensuring efficient oxygen delivery throughout the body. This highly regulated process begins in the bone marrow with hematopoietic stem cells, which differentiate through multiple progenitor stages into mature erythrocytes. Erythropoiesis is primarily regulated by EPO, a glycoprotein hormone produced predominantly by the kidneys in response to tissue hypoxia. EPO stimulates proliferation, differentiation, and survival of erythroid precursors, thereby maintaining hemoglobin homeostasis^[23,24]^. Aging introduces complex challenges to erythropoietic efficiency. Renal senescence often reduces EPO production, while age-related chronic inflammation – characterized by elevated cytokines such as IL-6 and TNF-α – impairs iron utilization and inhibits erythroid progenitor proliferation, a phenomenon referred to as “anemia of inflammation” or “anemia of aging.” Nutritional deficiencies in iron, vitamin B12, and folate further exacerbate the decline in RBC production in older adults. Consequently, age-associated reductions in erythropoiesis contribute to lower hemoglobin levels, diminished oxygen delivery, and increased susceptibility to fatigue, cognitive decline, and cardiovascular stress^[25,26]^.

Mechanistically, impaired erythropoiesis interacts with systemic aging pathways. Reduced RBC production limits oxygen availability to metabolically active tissues, leading to hypoxia-induced oxidative stress and activation of HIFs. While transient HIF activation can enhance adaptive responses, chronic stimulation promotes inflammation, vascular remodeling, and mitochondrial dysfunction, accelerating cellular senescence. Furthermore, age-related changes in bone marrow architecture, including reduced hematopoietic stem cell reserves and altered stromal support, impair erythroid differentiation and contribute to hematologic frailty^[27,28]^. Genetic factors and hemoglobin variants also modulate erythropoietic responses with age. Polymorphisms affecting globin gene expression, EPO receptor sensitivity, or iron metabolism can influence both baseline hemoglobin levels and adaptive erythropoiesis, thereby impacting longevity. Environmental factors, including altitude exposure and chronic hypoxia, further interact with erythropoietic mechanisms, often inducing compensatory polycythemia but potentially increasing cardiovascular risk if sustained^[29]^. Clinically, understanding the dynamics of erythropoiesis in aging is essential for predicting and managing anemia, optimizing hemoglobin levels, and improving systemic oxygen delivery. Interventions that support erythropoietic efficiency – ranging from nutritional supplementation to targeted pharmacologic strategies like EPO analogues – may mitigate age-related declines in hemoglobin and enhance both health span and lifespan^[30]^.

### Oxidative stress and hemoglobin integrity

Hemoglobin, while essential for oxygen transport, exists at the interface of oxygen delivery and ROS generation, making it both a facilitator of life and a potential contributor to cellular damage. The iron within the heme moiety of hemoglobin can participate in redox reactions, leading to the formation of superoxide anions, hydrogen peroxide, and other ROS under conditions of oxidative stress. While ROS are necessary for signaling and immune defense, excessive production can overwhelm antioxidant defenses, damaging hemoglobin itself, destabilizing erythrocyte membranes, and impairing oxygen-carrying capacity^[31,32]^. Aging exacerbates this vulnerability. Chronic low-grade inflammation, mitochondrial dysfunction, and accumulated cellular damage enhance systemic oxidative stress, which, in turn, accelerates hemoglobin oxidation. Oxidized hemoglobin can form methemoglobin, which is incapable of binding oxygen, and can trigger further oxidative reactions within erythrocytes. This cascade contributes to reduced RBC lifespan, anemia, and impaired tissue oxygenation – factors that negatively influence health span and longevity^[33,34]^.

Hemoglobin integrity is also influenced by genetic variants and post-translational modifications. For example, sickle cell hemoglobin (HbS) and other structurally altered hemoglobins are more prone to oxidative denaturation and membrane damage. Glycation of hemoglobin, as observed in diabetes, further compromises its functional capacity and enhances ROS-mediated injury, linking metabolic dysregulation with impaired oxygen transport and accelerated aging processes^[35]^. Antioxidant systems, including glutathione, catalase, and superoxide dismutase, play a critical role in maintaining hemoglobin integrity. These defenses buffer ROS and protect hemoglobin and RBC membranes from oxidative damage. However, age-related declines in antioxidant capacity can reduce this protection, creating a vicious cycle where hemoglobin dysfunction contributes to further oxidative stress and systemic aging^[36]^. Clinically, maintaining hemoglobin integrity has important implications. Strategies that reduce oxidative stress – such as adequate micronutrient intake (iron, selenium, and vitamins C and E), lifestyle interventions, and management of comorbidities – can preserve hemoglobin function, enhance oxygen delivery, and support cellular resilience. Understanding the interplay between hemoglobin, oxidative stress, and aging provides a mechanistic framework for interventions aimed at promoting healthy lifespan^[37]^.

## Hemoglobin and tissue oxygenation in aging

Efficient tissue oxygenation is fundamental to maintaining cellular metabolism, organ function, and overall health throughout the lifespan. Hemoglobin, by transporting oxygen from the lungs to peripheral tissues, serves as the central mediator of this process. In aging, however, multiple factors converge to compromise hemoglobin-mediated oxygen delivery, with significant consequences for systemic physiology and longevity^[38]^. As individuals age, hemoglobin levels often decline due to diminished erythropoiesis, nutritional deficiencies, chronic inflammation, and comorbid conditions such as renal impairment or cardiovascular disease. Reduced hemoglobin concentration directly limits the oxygen-carrying capacity of blood, impairing the delivery of oxygen to metabolically active tissues. This hypoxic stress can activate HIFs, which initially promote adaptive responses, including angiogenesis and erythropoietin production. Yet chronic HIF activation, as observed in prolonged anemia or systemic hypoxia, contributes to inflammatory signaling, tissue remodeling, and mitochondrial dysfunction, accelerating cellular senescence and organ aging^[39]^.

Tissue oxygenation is further influenced by hemoglobin affinity for oxygen, which is modulated by allosteric effectors such as 2,3-BPG, pH, and carbon dioxide levels. Aging-associated metabolic shifts, including acidosis and reduced red cell 2,3-BPG content, can alter oxygen release at the tissue level, compromising cellular respiration even when total hemoglobin levels are within normal ranges. This phenomenon underscores the importance of assessing both hemoglobin concentration and functional oxygen delivery when evaluating age-related physiological decline^[40]^. Moreover, demographic and environmental factors impact hemoglobin-mediated tissue oxygenation. Sex-specific differences, racial and ethnic variations, altitude exposure, and lifestyle factors all influence baseline hemoglobin levels and oxygen-carrying capacity, affecting tissue perfusion and resilience to age-related stressors. In addition, pathological states such as cardiovascular disease, diabetes, or chronic pulmonary conditions can synergistically reduce oxygen availability, further linking hemoglobin dysfunction to accelerated aging^[41]^.

From a clinical perspective, maintaining optimal hemoglobin levels and ensuring effective tissue oxygenation are critical for preserving cognitive function, physical performance, and cardiovascular health in older adults. Interventions targeting anemia, promoting erythropoietic efficiency, optimizing 2,3-BPG levels, and mitigating comorbidities can collectively enhance oxygen delivery, slow functional decline, and support longevity^[42]^.

### Influence of comorbid conditions on hemoglobin and longevity

Hemoglobin levels and functionality are profoundly influenced by a range of comorbid conditions, which can in turn affect systemic oxygen delivery, metabolic homeostasis, and aging trajectories. Understanding these interactions is critical for elucidating how hemoglobin dynamics contribute to longevity. The kidneys are the primary site of EPO production, the hormone that drives erythropoiesis. In CKD, reduced nephron function leads to impaired EPO synthesis, resulting in anemia of renal origin. This decrease in hemoglobin limits oxygen availability to tissues, exacerbating oxidative stress, mitochondrial dysfunction, and organ senescence. CKD-related anemia is strongly associated with increased cardiovascular morbidity and mortality, underscoring its relevance to lifespan outcomes^[43]^.

Conditions such as heart failure, ischemic heart disease, and atherosclerosis compromise tissue perfusion and oxygen delivery. Suboptimal hemoglobin levels further impair oxygen transport in these patients, amplifying hypoxic stress and triggering maladaptive responses such as chronic inflammation, endothelial dysfunction, and accelerated vascular aging. Maintaining adequate hemoglobin in cardiovascular disease is therefore essential for preserving functional capacity and reducing mortality risk^[44]^. Aging is often accompanied by low-grade chronic inflammation, sometimes referred to as “inflammaging.” Pro-inflammatory cytokines, including IL-6, TNF-α, and interferons, disrupt iron metabolism and inhibit erythroid progenitor differentiation, leading to anemia of inflammation. This inflammatory milieu not only reduces hemoglobin levels but also promotes oxidative damage and cellular senescence, creating a feedback loop that accelerates physiological aging^[45]^.

Iron, vitamin B12, and folate deficiencies are common in older adults and critically impact hemoglobin synthesis. Inadequate nutrient availability limits erythropoiesis, reduces hemoglobin concentration, and compromises oxygen delivery. Chronic nutrient deficiencies can thereby contribute to fatigue, cognitive impairment, and reduced organ resilience, all of which influence lifespan and health span^[46]^. Diabetes mellitus and obesity exert complex effects on hemoglobin function. Hyperglycemia leads to glycation of hemoglobin, reducing its oxygen-carrying efficiency and enhancing oxidative stress. Obesity-related inflammation further disrupts erythropoiesis and iron homeostasis, linking metabolic dysregulation to impaired hemoglobin integrity and accelerated aging^[47]^.

Comorbid conditions rarely act in isolation. The combined effects of renal dysfunction, cardiovascular disease, chronic inflammation, nutritional deficiencies, and metabolic disorders create compounded risks for impaired hemoglobin regulation, tissue hypoxia, and systemic oxidative stress. This convergence contributes to reduced physiological reserve, increased vulnerability to stressors, and ultimately, shortened lifespan^[48]^. Clinically, these insights underscore the importance of holistic management strategies that address both underlying comorbidities and hemoglobin optimization. Interventions may include targeted nutritional support, correction of iron and vitamin deficiencies, management of chronic inflammation, and careful monitoring of erythropoietic function. By mitigating the deleterious effects of comorbid conditions on hemoglobin and tissue oxygenation, it may be possible to preserve functional capacity and enhance longevity in aging populations^[49]^.

## Demographic and environmental variations in hemoglobin and longevity

Hemoglobin levels and their functional impact on tissue oxygenation and longevity are modulated by demographic and environmental factors. Understanding these variations is essential for accurate clinical assessment, personalized interventions, and interpretation of hemoglobin-related outcomes in aging populations.

### Sex differences

Hemoglobin concentrations are generally higher in men than in women, largely due to androgenic stimulation of erythropoiesis and differences in iron metabolism. Women, particularly postmenopausal women, may experience declines in hemoglobin associated with hormonal changes, nutritional deficiencies, or chronic disease, which can influence oxygen delivery and accelerate age-related functional decline. Sex-specific considerations are therefore critical when evaluating hemoglobin thresholds and tailoring interventions aimed at preserving longevity^[50]^.

### Racial and ethnic variations

Hemoglobin levels vary across racial and ethnic groups due to genetic, nutritional, and environmental factors. For example, individuals of African descent often have lower baseline hemoglobin but may tolerate these levels without apparent functional compromise, potentially due to adaptive erythropoietic responses or hemoglobin variants. Conversely, populations with hemoglobinopathies, such as sickle cell trait or thalassemia, exhibit altered hemoglobin structure and function, impacting oxygen transport and systemic aging. Recognition of these variations is important for avoiding misdiagnosis, optimizing treatment, and understanding population-specific longevity risks^[45,46]^.

### Altitude and environmental exposure

Chronic exposure to high altitude induces compensatory polycythemia through hypoxia-driven erythropoiesis, increasing hemoglobin levels to maintain tissue oxygenation. While this adaptation supports oxygen delivery in hypoxic environments, sustained elevations in hemoglobin can increase blood viscosity and cardiovascular risk, particularly in older adults. Environmental factors such as chronic air pollution, smoking, and hypoxic conditions also modulate hemoglobin levels and oxidative stress, influencing systemic aging pathways^[47,48]^.

### Implications for clinical assessment and aging

Demographic and environmental variations necessitate individualized interpretation of hemoglobin measurements. Clinicians must consider sex, race/ethnicity, altitude, and lifestyle factors when assessing anemia, polycythemia, or functional oxygen delivery. Personalized approaches to monitoring and intervention – such as adjusting hemoglobin targets, supplementing nutrients, or mitigating environmental exposures – can enhance tissue oxygenation, preserve physiological resilience, and promote longevity^[49,50]^.

## Clinical and therapeutic implications

Understanding the interplay between hemoglobin physiology, oxygen delivery, and systemic aging provides valuable insights for clinical practice and interventions aimed at promoting health span and longevity. Hemoglobin levels serve as a readily measurable biomarker of systemic oxygenation, metabolic resilience, and overall physiological integrity. Both low and high hemoglobin concentrations are associated with adverse outcomes in aging populations, including cognitive decline, cardiovascular events, reduced physical performance, and increased mortality. Thus, maintaining hemoglobin within an optimal physiological range is critical for healthy aging^[43,44]^. Age-related anemia, often multifactorial, requires comprehensive evaluation. Nutritional deficiencies (iron, vitamin B12, folate), chronic kidney disease, inflammatory states, and hematologic disorders should be systematically assessed. Interventions such as targeted supplementation, correction of underlying deficiencies, and judicious use of erythropoiesis-stimulating agents (ESAs) can restore hemoglobin levels, enhance oxygen delivery, and mitigate functional decline. Clinical guidelines now emphasize individualized hemoglobin targets based on comorbidities and functional status rather than universal thresholds, reflecting the nuanced impact of hemoglobin on aging outcomes^[45,46]^.

Conversely, elevated hemoglobin, as seen in polycythemia vera or chronic hypoxia, increases blood viscosity, predisposing to thrombotic events and vascular complications. Therapeutic strategies, including phlebotomy, cytoreductive agents, and management of contributing conditions (e.g., chronic hypoxia from pulmonary disease), are critical for minimizing cardiovascular risk while preserving oxygen-carrying capacity^[47]^. Physical activity, high-altitude acclimatization, and dietary optimization influence hemoglobin production and functional oxygen delivery. Exercise enhances erythropoiesis, increases 2,3-BPG levels, and improves microvascular perfusion, collectively supporting tissue oxygenation and metabolic efficiency. Similarly, nutritional strategies rich in iron, antioxidants, and cofactors (vitamin B12, folate, selenium, vitamin C) help maintain hemoglobin integrity and protect against oxidative damage^[48]^.

Demographic, genetic, and environmental factors – including sex, race, altitude, and hemoglobin variants – modulate hemoglobin dynamics and aging trajectories. Personalized assessments, integrating these factors with comorbidity profiles, allow for targeted interventions that optimize oxygen delivery and systemic resilience, thereby enhancing health span^[49]^. Emerging therapies targeting erythropoietin signaling, redox balance, and mitochondrial function hold promise for extending health span by supporting hemoglobin function. Monitoring hemoglobin as part of a broader biomarker panel for aging could also provide early warning for functional decline and guide preventive strategies^[11,50]^.

## Key contributions and learning points

This narrative review provides a comprehensive synthesis of the emerging evidence linking hemoglobin physiology with human longevity, offering several novel insights that extend beyond the existing literature. First, it highlights hemoglobin not merely as an oxygen transporter but as a central regulator of systemic health, integrating metabolic efficiency, redox homeostasis, immune function, and tissue resilience. By examining hemoglobin through the lens of aging biology, the review emphasizes its role in mitigating oxidative stress, modulating cellular senescence, and influencing mitochondrial function – mechanisms that are increasingly recognized as critical determinants of lifespan.

Second, the review critically evaluates how deviations from optimal hemoglobin levels – both anemia and polycythemia – affect health span and lifespan. It underscores the interplay between hemoglobin and comorbid conditions such as chronic kidney disease, nutritional deficiencies, inflammation, and cardiovascular dysfunction, providing a nuanced understanding of how systemic physiology mediates longevity outcomes. Unlike prior reviews that often treat hemoglobin changes in isolation, this work contextualizes these variations within broader age-related pathophysiology, offering a holistic perspective.

Third, this review integrates mechanistic insights with practical clinical implications. It delineates the influence of hemoglobin variants, erythropoietin signaling, and modulators like 2,3-bisphosphoglycerate (2,3-BPG) on oxygen delivery and tissue metabolism, translating complex molecular processes into clinically relevant observations. Furthermore, it highlights demographic and environmental influences, including sex, ethnicity, and altitude, emphasizing the importance of personalized assessments in predicting longevity and guiding interventions.

Finally, the review identifies actionable learning points for researchers and clinicians. These include the value of routine hemoglobin monitoring in older adults, the potential for nutritional and pharmacologic interventions to optimize hemoglobin levels, and the need for interdisciplinary approaches that link hematology, gerontology, and preventive medicine. By synthesizing existing evidence and highlighting gaps, this review provides a roadmap for future studies aimed at harnessing hemoglobin as a biomarker and potential therapeutic target for promoting healthy aging.

## Conclusion

Hemoglobin stands at the crossroads of molecular biology and human longevity, functioning not only as an oxygen transporter but as a critical determinant of systemic vitality and resilience. As this review has explored, its role extends far beyond erythrocytes and laboratory values – it interweaves with the aging process through mechanisms involving erythropoiesis, oxidative stress, vascular dynamics, and tissue oxygenation. The age-related decline in hemoglobin functionality and red cell integrity mirrors broader physiological changes, many of which contribute to frailty, diminished organ performance, and chronic disease progression in the elderly. It encourages clinicians and researchers alike to shift from a reactive to a proactive stance – recognizing subtle declines in hemoglobin performance as early warning signs and potential opportunities for therapeutic intervention. Advances in molecular therapies, precision diagnostics, and supportive lifestyle strategies offer promising avenues to preserve hemoglobin function and, by extension, improve quality of life in aging populations.

## References

[1] Albert P Katz A. Lifespan decoded: how to hack your biology for a longer, healthier life. Longerton LLC 2025.

[2] Rosove MH. The Function of Normal Human Hemoglobin. InLife’s Blood: The Story of Hemoglobin. Cham: 2024. Springer Nature Switzerland; 21–32.

[3] McClain R. Cheating Death: The New Science of Living Longer and Better. BenBella Books; 2023.

[4] Prasad P Mathew A Jose S. Fostering healthy longevity through regenerative and precision medicine: biodiversity’s vital role and equitable benefit sharing. In: InBiodiversity and Business. Cham: Springer; 2024:563–94.

[5] Tregoning JS. Live Forever: A Curious Scientist’s Guide to Wellness, Ageing and Death. Simon and Schuster; 2025.

[6] Farahani A Liu ZQ Ceballos EG. Cerebral blood perfusion across biological systems and the human lifespan bioRxiv. 2025;2025–02. doi: 10.1101/2025.02.05.636674.

[7] Ramakrishnan V. Why We Die: The New Science of Ageing and Longevity. Hachette UK; 2024.

[8] Mackieh R Al-Bakkar N Kfoury M. Unlocking the benefits of fasting: a review of its impact on various biological systems and human health. Curr Med Chem 2024;31:1781–803. 38018193 10.2174/0109298673275492231121062033

[9] Agha RA Mathew G Rashid R. TITAN Group. Transparency in the reporting of Artificial Intelligence – the TITAN Guideline. Premier Journal of Science 2025;10:100082.

[10] Raza U Tang X Liu Z. SIRT7: the seventh key to unlocking the mystery of aging. Physiol Rev 2024;104:253–80. 37676263 10.1152/physrev.00044.2022 PMC11281815

[11] Ren J Wang Z Zhang Y. Is hemoglobin concentration a linear predictor of mortality in older adults from chinese longevity regions? Front Public Health 2021;9:787935. 34912772 10.3389/fpubh.2021.787935 PMC8666873

[12] Yu F Chen C Liu W. Longevity humans have youthful erythrocyte function and metabolic signatures. Aging Cell 2025;24:e14482. 39924931 10.1111/acel.14482 PMC12074018

[13] Rezapour M Shadpour P Karimi A. Inverse effects of anemia and diabetes mellitus on non-cuffed central venous catheters longevity. Iran J Vasc Surg Endovasc Ther 2021;1:31–39.

[14] Yang X Zhao B Wang J. Red blood cell lifespan in long-term hemodialysis patients treated with roxadustat or recombinant human erythropoietin. Ren Fail 2021;43:1428–36. 34657570 10.1080/0886022X.2021.1988968 PMC8525968

[15] Chen WL Nishita Y Nakamura A. Hemoglobin concentration is associated with the hippocampal volume in community-dwelling adults. Arch Gerontol Geriatr 2022;101:104668. 35248921 10.1016/j.archger.2022.104668

[16] Wang G Huang Y Zhang N. Hydrogen sulfide is a regulator of hemoglobin oxygen-carrying capacity via controlling 2, 3-BPG production in erythrocytes. Oxid Med Cell Longev 2021;2021:8877691. 33628390 10.1155/2021/8877691 PMC7896853

[17] Geetha IIIS Verma N Chakole V. A comprehensive review of extra corporeal membrane oxygenation: the lifeline in critical moments. Cureus 2024;16:e53275. 10.7759/cureus.53275 PMC10905309 38435953

[18] Rabcuka J Smethurst PA Dammert K. Assessing the kinetics of oxygen-unloading from red cells using flowScore, a flow-cytometric proxy of the functional quality of blood. EBioMedicine 2025;111:105498. 10.1016/j.ebiom.2024.105498 PMC11730303 39674089

[19] Ilmiyani SN Yanti EM Siswari BD. The effect of delayed cord clamping (DCC) on haemoglobin levels and oxygen saturation levels in newborns. Babali Nursing Research 2023;4:420–30.

[20] Obeagu GU Altraide BO, and Obeagu EI. Iron deficiency anemia in pregnancy and related complications with specific insight in Rivers State, Nigeria: a narrative review. Ann Med Surg 2025;87:3435–44. 10.1097/MS9.0000000000003224 PMC12140781 40486642

[21] Yanti LN Rofinda ZD Yusri E. Impact of donation frequency on iron stores and hemoglobin levels in regular blood donors. Bioscientia Med. J. Biomed. Transl. Res 2025;9:7401–12.

[22] Ma H Wang Z Geng J. Effects of integrated blood purification on haemodynamics and oxygen metabolism in children with severe sepsis. Front Med (Lausanne) 2024;11:1400154. 39564495 10.3389/fmed.2024.1400154 PMC11573546

[23] Shah A Srivastava S Chaturvedi CP. Biomolecular Components of Blood and Their Role in Health and Diseases. InClinical Applications of Biomolecules in Disease Diagnosis: A Comprehensive Guide to Biochemistry and Metabolism. Singapore: 2024. Springer Nature Singapore; 289–322.

[24] Zheng H Jiang L Tsuduki T. Embryonal erythropoiesis and aging exploit ferroptosis. Redox Biol 2021;48:102175. 34736120 10.1016/j.redox.2021.102175 PMC8577445

[25] Mbiandjeu SC Siciliano A Mattè A. Nrf2 plays a key role in erythropoiesis during aging. Antioxidants 2024;13:454. 38671902 10.3390/antiox13040454 PMC11047311

[26] Chen S Liu Y Cai L. Erythropoiesis changes with increasing age in the elderly Chinese. Int J Lab Hematol 2021;43:1168–73. 34125997 10.1111/ijlh.13615

[27] Bruserud Ø Vo AK Rekvam H. Hematopoiesis, inflammation and aging – the biological background and clinical impact of anemia and increased C-reactive protein levels on elderly individuals. J Clin Med 2022;11:706. 35160156 10.3390/jcm11030706 PMC8836692

[28] Wacka E Wawrzyniak-Gramacka E Tylutka A. The role of inflammation in age-associated changes in red blood system. Int J Mol Sci 2023;24:8944. 37240288 10.3390/ijms24108944 PMC10219258

[29] Caulier AL Sankaran VG. Molecular and cellular mechanisms that regulate human erythropoiesis. Blood. The Journal of the American Society of Hematology 2022;139:2450–59. 10.1182/blood.2021011044 PMC9029096 34936695

[30] Antonelou MH D’Alessandro A Kriebardis AG. In sickness and in health: erythrocyte responses to stress and aging. Int J Mol Sci 2022;23:6957. 35805962 10.3390/ijms23136957 PMC9267024

[31] Larsson SM Ulinder T Rakow A. Hyper high haemoglobin content in red blood cells and erythropoietic transitions postnatally in infants of 22 to 26 weeks’ gestation: a prospective cohort study. Archives of Disease in Childhood-Fetal and Neonatal Edition 2023;108:612–16. 37169579 10.1136/archdischild-2022-325248 PMC10646872

[32] Orrico F Laurance S Lopez AC. Oxidative stress in healthy and pathological red blood cells. Biomolecules 2023;13:1262. 37627327 10.3390/biom13081262 PMC10452114

[33] Obeagu EI Igwe MC Obeagu GU. Oxidative stress’s impact on red blood cells: unveiling implications for health and disease. Medicine (Baltimore) 2024;103:e37360. 38428906 10.1097/MD.0000000000037360 PMC10906601

[34] Kosmachevskaya OV Novikova NN Topunov AF. Carbonyl stress in red blood cells and hemoglobin. Antioxidants 2021;10:253. 33562243 10.3390/antiox10020253 PMC7914924

[35] Fujii J Homma T Kobayashi S. Erythrocytes as a preferential target of oxidative stress in blood. Free Radic Res 2021;55:781–99. 10.1080/10715762.2021.1873318 33427524

[36] Alayash AI. βCysteine 93 in human hemoglobin: a gateway to oxidative stability in health and disease. Lab Invest 2021;101:4–11. 32980855 10.1038/s41374-020-00492-3

[37] Morales NP Rodrat S Piromkraipak P. Iron chelation therapy with deferiprone improves oxidative status and red blood cell quality and reduces redox-active iron in β-thalassemia/hemoglobin E patients. Biomed Pharmacother 2022;145:112381. 34736078 10.1016/j.biopha.2021.112381

[38] Zhuge Z Haworth SM Nihlén C. Red blood cells from endothelial nitric oxide synthase-deficient mice induce vascular dysfunction involving oxidative stress and endothelial arginase I. Redox Biol 2023;60:102612. 36681048 10.1016/j.redox.2023.102612 PMC9868875

[39] Lugthart G Verweij EJ Harteveld CL. Suppression of Hb Bart’s to improve tissue oxygenation and fetal development in homozygous alpha-thalassemia. Am J Hematol 2024;99:1613–15. 38655712 10.1002/ajh.27344

[40] Kalteren WS Verhagen EA Mintzer JP. Anemia and red blood cell transfusions, cerebral oxygenation, brain injury and development, and neurodevelopmental outcome in preterm infants: a systematic review. Front Pediatr 2021;9:644462. 33718309 10.3389/fped.2021.644462 PMC7952449

[41] Karlas A Masthoff M Kallmayer M. Multispectral optoacoustic tomography of peripheral arterial disease based on muscle hemoglobin gradients – a pilot clinical study. Ann Transl Med 2021;9:36. 33553329 10.21037/atm-20-3321 PMC7859778

[42] Aref MH Sharawi AA El-Sharkawy YH. Delineation of the arm blood vessels utilizing hyperspectral imaging to assist with phlebotomy for exploiting the cutaneous tissue oxygen concentration. Photodiagnosis Photodyn Ther 2021;33:102190. 33508500 10.1016/j.pdpdt.2021.102190

[43] Blume SY Garg A Martí-Mateos Y. HypoxyStat, a small-molecule form of hypoxia therapy that increases oxygen-hemoglobin affinity. Cell 2025;188:1580–88. 39965572 10.1016/j.cell.2025.01.029 PMC12186697

[44] Oyedeji CI Artz AS Cohen HJ. How I treat anemia in older adults. Blood 2024;143:205–13. 36827619 10.1182/blood.2022017626 PMC10808247

[45] Xu JZ Thein SL. Revisiting anemia in sickle cell disease and finding the balance with therapeutic approaches. Blood. The Journal of the American Society of Hematology 2022;139:3030–49. 10.1182/blood.2021013873 PMC9121844 35587865

[46] Pires IS Berthiaume F Palmer AF. Engineering therapeutics to detoxify hemoglobin, heme, and iron. Annu Rev Biomed Eng 2023;25:1–21. 37289555 10.1146/annurev-bioeng-081622-031203

[47] Reisz JA Earley EJ Nemkov T. Arginine metabolism is a biomarker of red blood cell and human aging. Aging Cell 2025;24:e14388. 39478346 10.1111/acel.14388 PMC11822668

[48] Vallelian F Buehler PW Schaer DJ. Hemolysis, free hemoglobin toxicity, and scavenger protein therapeutics. Blood. The Journal of the American Society of Hematology 2022;140:1837–44. 10.1182/blood.2022015596 PMC10653008 35660854

[49] Obeagu EI. Maximizing longevity: erythropoietin’s impact on sickle cell anaemia survival rates. Ann. Med. Surg 2024;86:1570–74. 10.1097/MS9.0000000000001763 PMC10923353 38463100

[50] Yang J Zhou C Li HJ. Effects of lifestyle and its interaction with anemia on cognitive function in older adults: a longitudinal study. Psych J 2024;13:242–51. 38105563 10.1002/pchj.712 PMC10990814

